# An Overview of the Nutritional Requirements of Honey Bees (*Apis mellifera* Linnaeus, 1758)

**DOI:** 10.3390/insects16010097

**Published:** 2025-01-18

**Authors:** Leticia S. Ansaloni, Janja Kristl, Caio E. C. Domingues, Aleš Gregorc

**Affiliations:** Faculty of Agriculture and Life Sciences, University of Maribor, Pivola 10, 2311 Hoče, Slovenia; janja.kristl@um.si (J.K.); cecdomingues@gmail.com (C.E.C.D.); ales.gregorc@um.si (A.G.)

**Keywords:** development, health, nectar, nutrition, pollen

## Abstract

Honey bees are known for their wide global distribution, their ease of handling, and their economic and ecological value. However, they are often exposed to a wide variety of stress factors. Therefore, it is essential for beekeepers to maintain healthy bee colonies. In this context, a balanced diet is recommended to support the growth of strong and healthy honey bee colonies. The purpose of this review is therefore to provide an overview of the nutritional requirements of *Apis mellifera* and their importance for the maintenance of healthy bee colonies. An adequate diet includes the consumption of sufficient amounts of proteins, carbohydrates, lipids, amino acids, vitamins, minerals, water, and essential sterols, and a diet based on multi-floral pollen is desirable. However, when honey bee colonies are located near agroecosystems with lower resource diversity, both brood rearing and colony longevity may decrease, making them more susceptible to parasites and diseases. On the other hand, efforts have been made to improve the health of honey bee colonies with the help of nutritional supplements consisting of a variety of components. Nevertheless, studies have shown that even with these supplements, a lack of nutrients can still be an issue for honey bee colonies. Furthermore, future research should focus on identifying nutritional supplements that can better replicate natural diet diversity and assessing long-term effects on honey bee colony resilience, especially in low-flowering areas. This review discusses the interaction between nutrient requirements and the effects of supplements on colony health.

## Simple Summary

It is well documented in the literature that bees have been facing numerous stressors in recent decades. Although honey bees are well managed worldwide, they have not been unaffected by these challenges. Honey bees are important for pollination in various crops, for the beekeeping sector and for the natural habitats as well. However, to perform this role effectively, they require proper nutrition to support their healthy growth and that of the colony. In the current review, we broadly discuss the basic nutritional needs of honey bees, such as the consumption of proteins, carbohydrates, lipids, amino acids, vitamins, minerals, water, and other essential components. Additionally, we also discuss the use of nutritional supplements aimed at supporting the health and strengthening of the colony, and how, despite these efforts, inadequate nutrition can still be a problem for honey bee colonies.

## 1. Introduction

Pollination stands out as one of the most essential ecosystem services. According to Ollerton et al. [1], it is estimated that approximately 87.5% of all flowering plant species depend on animal pollination. Bees provide valuable pollination services to a wide range of crops and native flora worldwide [2,3,4]. Among the diversity of bee species, with over 20,000 already described worldwide [5], the honey bee (*Apis mellifera* Linnaeus, 1758) distinguishes itself globally for its substantial economic and ecological value, derived from the essential role it plays in pollination [6]. This species holds significant economic importance by not only increasing production and quality in agricultural fields but also through the commercialization of bee products like apitoxin (bee venom), honey, propolis, royal jelly, and wax [3,7].

According to Ilyasov et al. [8], although there are differences in taxonomic revisions, thirty-three distinct honey bee subspecies are currently found in Europe, Africa, and Western Asia and the Middle East. Moreover, due to the Africanization process resulting from the intercross of European and African subspecies, the hybrid Africanized *A. mellifera* has also spread throughout the Americas [9,10]. Depending on the subspecies, honey bees may exhibit less defensive behavior and an enhanced ability to adapt to colder climates, as observed in *A. m. carnica* [11], or more defensive behavior, as found in *A. m. scutellata* [12], as well as better adaptability to warm environments, as described for *A. m. ligustica* [13]. Nevertheless, the honey bees have a strong foraging orientation and exhibit high performance in honey production [14,15].

Many studies have been conducted within breeding programs for *A. mellifera* to improve desirable honey bee traits, aiming to obtain healthier and more productive colonies [11,16,17,18,19,20]. These efforts also include enhancing resistance to Varroa [21,22,23], and screening for the presence of viruses during queen rearing [24,25,26].

However, honey bee colony mortality has impacted beekeepers worldwide over the past decades [27], calling for effective mitigation strategies. Goulson et al. [28] highlighted multiple factors associated with the decline in bees, such as habitat loss, parasites and disease, pesticides, monotonous diets, shipping fever, competition, and climate change. It is currently known that the health risks for honey bees increase when two or more stressors are combined [29]. Nowadays, it is crucial for farmers and beekeepers worldwide to maintain healthy honey bee colonies, especially since these colonies are often exposed to stressors and are frequently located near agroecosystems and natural forest areas [30].

Winkler et al. [31] reported that global land use has increased more than previously estimated in recent decades, which may be linked to global trade in agricultural production. Similarly, large-scale monoculture has negatively impacted apiculture by reducing honey yield, as described by de Groot et al. [32]. As mentioned before, monotonous diets are one of the stress factors for bees, and honey bees are highly dependent on the availability and diversity of floral resources, which are crucial for the development and survival of their colonies [33]. This combination of diverse floral sources is important because, depending on the species, these sources can vary in protein, amino acid, lipid, carbohydrates, vitamins, and other nutritional contents [34]. Concerns about honey bees receiving an inadequate diet have driven the development of protein supplements, and efforts have been made to study the effectiveness of these supplements on colony health [35,36,37]. Brodschneider and Crailsheim [34] suggested that a balanced diet is necessary to maintain well-fed and healthy colonies. Similarly, queens, workers, and drones have different physiology and nutritional demands during their development and throughout their lives [38], which further highlights the importance of nutritional diversity.

Based on the aforementioned considerations and the importance of nutrition for healthy colonies, this study aims to provide an overview of the diet and nutritional requirements of *A. mellifera*, as well as how these factors influence the development of larvae and adult, including the use of nutritional supplements.

## 2. Nutritional Requirements for Honey Bees

*A. mellifera* is the most frequently managed bee species globally [39] and is found on all continents except Antarctica. The honey bee has been used as a model organism for the study of various topics due to the extensive knowledge of its biology [40]. In this regard, research in bee nutrition has made significant progress over time, allowing a better understanding of the specific nutritional requirements of *A. mellifera* [34,41]. The essential nutrients required for the growth, development, and survival of honey bee colonies are highlighted below.

### 2.1. Proteins

Pollen is the main source of protein for honey bees, which they collect from a wide variety of plants [3]. Studies have shown that the protein content of pollen varies depending on the plant species [42]. Radev [43] determined the crude protein content in mixed pollen species collected during different harvesting periods (April to September) in Bulgaria and found a range of 11.5% to 27.4%, while a literature review by Roulston et al. [44] found a wider range of protein content from 2.5% to 61% in the pollen of 377 plant species from 93 plant families. In this regard, the quality and diversity of pollen are important for honey bee physiology and health [33], as the protein content and amino acid profile can vary, and a lack of pollen diversity can impair development and leave the colony more susceptible to pathogens and diseases [45]. A diet consisting of multi-flower pollen is preferable [46], as balanced nutrition more effectively ensures the proper growth, development, and overall health of honey bee colonies.

The amount of pollen collected by a honey bee colony can be influenced by different factors, including climatic conditions, season, colony size, health status, and the availability and diversity of floral sources [47,48], which also affect protein levels in the colony. Wille et al. [49] described a range of 10–26 kg of pollen collected per year, whereas Avni et al. [50] reported 16.8 kg of pollen per colony as an overall annual mean, with the variations depending on sites and seasons. However, higher values, with pollen collection reaching up to 55 kg per year, have already been reported in the literature [51]. The nutritional value of pollen is a valuable source of amino acids, lipids, carbohydrates, minerals, vitamins, and essential sterols [52]. These components are closely linked to the growth, reproduction, and productivity of *A. mellifera* colonies [53]. According to Radev [54], honey bee colony reproduction increases in the presence of pollen with high protein content, specifically over 21 and 27%. In contrast, if pollen access is reduced, brood rearing and colony lifespan may decrease [55].

The protein requirements of adult bees and honey bees’ larvae are different. A study by Crailsheim et al. [56] showed that the daily pollen consumption of an adult honey bee was 3.4–5.4 mg, depending on age, while the entire colony consumed between 13.4 and 17.8 kg of pollen annually. In contrast, Hrassnigg and Crailsheim [38] emphasized that larvae require a higher protein intake and need to consume 25–37.5 mg per reared larva (125–187.5 mg of pollen with a protein content of about 20%). According to Li et al. [57], a higher protein content in the larval diet can increase worker longevity, possibly due to the upregulation of genes related to antioxidant activity. A pollen-based diet is also associated with lower sensitivity to pesticides [58]. In addition, honey bees use pollen to make bee bread, a food consisting of a mixture of pollen, regurgitated nectar, and enzymes secreted by their glands [59]. Bee bread provides energy, has a higher nutritional value than pollen, and contains bioactive compounds such as phenolics and flavonoids, which exhibit antioxidant and antimicrobial activity [59,60]. According to Kieliszek et al. [59], it is more digestible for bees due to the presence of amino acids and serves as the main food source for larvae and young bees. However, the potential of bee bread has not yet been explored, and there is little relevant literature in comparison to protein feeds.

The use of protein-supplemented diets in honey bee colonies has increased among beekeepers worldwide in recent years [37,61]. DeGrandi-Hoffman et al. [62] observed that a commercial diet with 16.5% protein and 66% carbohydrate can stimulate the production of more brood. According to Zheng et al. [63], the reproduction rate of honey bees was maximized when they were fed with a crude protein content of 29.5–34.0%. Liquid protein supplementation (Hydroamin 30^®^—Bioal diluted at 4% in one liter of sucrose–water syrup 2:1 m/v) improved bee health and decreased post-winter mortality, as highlighted by García-Vicente et al. [64]. Given these findings, beekeepers should consider adding protein supplements to the diet during pollen shortages. On the other hand, DeGrandi-Hoffman et al. [65] demonstrated that natural forage was more effective than protein supplements in reducing pathogen load and resulted in higher over-winter survival, which was related to the greater digestion of pollen proteins. The authors suggest that the protein supplements were less digestible than pollen because the protein sources (soy, barley flour, and eggs) are not part of the bees’ diet. However, additional research is required to evaluate the effects of supplemented protein diets. Furthermore, bees are no longer finding the same resources that were available to them in previous years, which is why fructose and sucrose syrups are being more widely used. Studies have reported the impacts of these changes on bee nutrition and health [66,67].

### 2.2. Carbohydrates

Nectar is the most important source of carbohydrates (sucrose, glucose, and fructose) for honey bees, and flowers are their primary natural source [34,68]. Similar to the protein content in pollen, the carbohydrate ratio in nectar also varies depending on the plant species and its structural characteristics (e.g., flower shape and nectary structure) [69,70]. Additionally, honeydew, a sugary excretion produced by many different sap-feeding insects, serves as another important source of carbohydrates for honey bees [71].

Carbohydrates are an essential source of energy, providing the necessary fuel for foraging and internal colony activities [72]. According to a systematic review performed by Pamminger et al. [73], the average sugar concentration in nectar from crops, weeds, and wild plants worldwide is 40%, with the optimal sugar concentration suggested to range from 35% to 65%, based on uptake measurements and theoretical considerations by Kim et al. [74]. The nutritional value of nectar is based on three primary sugars: sucrose, glucose, and fructose [69]. Other sugars occur in lower concentrations or are difficult to break down [75]. Additionally, some nectars can be toxic to honey bee if ingested [76], such as from *Sophora microphylla*, which contains alkaloids (e.g., cytisine) [77], and *Ochroma lagopus*, known for its alkaloid content (e.g., atropine and scopolamine) [78]. These alkaloids are known for their toxic effects on the nervous system, physiology, and behavior of honey bees. In addition, among toxic nectars, it is important to highlight those from plants that contain pyrrolizidine alkaloids (PAs), the toxicity of which to adult honey bees and brood has already been reported [79,80,81,82]. This has raised concerns due to its potential risks to human health, as PAs are currently under examination by the European Food Safety Authority (EFSA) because of their toxicity [83,84,85].

Paoli et al. [86] demonstrated that the nutritional demand for carbohydrates increases depending on the honey bees’ age, with foragers (older bees) consuming 60% more than younger bees aged 0–7 and 8–14 days. The authors estimated that the carbohydrate demand increases from two to five times for foragers. According to Couvillon et al. [87], the foraging distances of honey bees vary depending on the month and the type of forage available. Based on the analysis of 4562 waggle dances decoded from nectar foragers, the average foraging distance was 1408 m, with the maximum distance exceeding 5000 m. These long distances demand a high energy expenditure. Additionally, larvae also need carbohydrates for the development, with the estimated sugar requirement being 59.4 mg for workers (5 days) and 98.2 mg for drones (6.5 days) [51].

Nectar is important as the main source for making honey [34]. Honey bees process the nectar by adding enzymes from their salivary glands to break down complex sugars and reducing large amounts of water to prevent fermentation [75]. The processed nectar is stored in combs and serves as an essential source of food during floral resource scarcity [52]. According to Silva et al. [88], honey is primarily a blend of sugars, water, proteins, vitamins, organic acids, minerals, pigments, phenolic compounds, and volatile compounds. The main carbohydrates present in honey are fructose (38.2%) and glucose (31.3%) on average [89], although sucrose, maltose, and other sugars can also be found [88].

The use of carbohydrate supplementation is also a common practice in beekeeping [61,75,90,91,92]. Sammataro and Weiss [75] highlighted that colonies fed with sucrose syrup built more honeycomb, had a higher average mass of bees, and showed increased spring brood production compared to colonies fed with high-fructose corn syrup, which is commonly used as a source of bee feed. On the other hand, honey bees exhibited sublethal effects on stress proteins, such as superoxide dismutase, suggesting an impaired response to oxidative stress when fed with sucrose syrup and high-fructose corn syrup [91]. Wheeler et al. [90] suggested that a diet of 50% (*w*/*v*) honey provides more nutritional components compared to 50% high-fructose corn syrup or 50% sucrose, which are widely used food sources in apiculture. According to Frizzera et al. [93], since sugar supplementation has become an increasingly common practice among beekeepers, it is necessary to be aware about the potential side effects on honey bee health, including a decrease in bee survival.

### 2.3. Lipids

Honey bees obtain lipids from the pollen they collect [52]. According to the findings of Dobson [94], nonpolar lipids may be present in the pollenkitt, while polar lipids are found in the inner pollen fraction. Both types of lipids are important for honey bees. The lipid content and composition in pollen varies depending on the plant species [44,95]. Roulston and Cane [44] compiled published data for 62 plant species, with the lipid content ranging from 0.8% in *Eucalyptus marginata* (Myrtaceae) to 18.9% in *Taraxacum officinale* (Asteraceae).

Lipids are essential for physiological processes related to energy, metabolism, and regulation [96]. They serve as a significant energy source for honey bees, store energy reserves, and are key components of cell membrane structures [96,97]. Lipids are primarily stored in the fat body, with larvae having a greater amount of lipids compared to adult honey bees [98]. However, studies have also shown that young nurse bees store a large amount of lipids in their abdomens [99,100]. Conversely, the amount of abdominal lipids decreases with age [101], which could be related to foraging activity.

In addition, fatty acids are also important for honey bees and contribute to antimicrobial properties, cognitive functions, and reproduction [52,100,102]. Palmitic acid, linoleic acid (omega-6), and alpha-linolenic acid (omega-3) are the most commonly found fatty acids in pollen [102], and together with oleic acid, they are also present in the body lipids of drones, workers, and queens [103].

Compared to other types of dietary supplementations, there are few studies on the use of lipid diets. Nonetheless, research has demonstrated that brood production in honey bees increases when lipid extracts (e.g., pollen lipid fraction) are included in their diet [104,105]. According to Arien et al. [105], brood rearing was best with an 8% lipid diet, while survival was highest with a 4% lipid diet and an omega-6:3 ratio of 1. However, further studies are needed to understand the long-term effects of feeding lipid diets.

### 2.4. Amino Acids and Vitamins

Amino acids also contribute significantly to the nutritional needs of honey bees, with key functions in their metabolism, including immune response [106], phagostimulatory effects [107], and learning and memory [108], as well as serving as neurotransmitters and contributing to other essential processes [109]. Pollen is a valuable source of amino acids, especially exogenous ones [110]. According to De Groot [111], the amino acids arginine, histidine, lysine, phenylalanine, tryptophan, leucine, isoleucine, threonine, and valine are essential for the development of adult honey bee workers.

In addition to amino acids, vitamins are also essential nutrients for honey bees and can be found in pollen, nectar, honey, royal jelly, and other food sources [52]. The concentrations of various vitamins, including thiamine, riboflavin, niacin, pantothenic acid, pyridoxine, folic acid, cobalamin, biotin, vitamin E, and ascorbic acid, vary in pollen, royal jelly, and honey [112]. A study conducted by Elsayeh et al. [112] demonstrated that B-vitamins can play an important role in regulating the uptake of macronutrients, such as carbohydrates and essential amino acids in honey bees.

Royal jelly (RJ) is a highly nutritious secretion produced by young nurse worker bees through their hypopharyngeal and mandibular glands [113]. It serves as an essential food source for the development of the larvae, especially the queens [68]. The composition of RJ includes water, proteins, free amino acids, carbohydrates, lipids, and minerals [114]. According to Howe et al. [115], RJ contains 17 amino acids and five unidentified ninhydrin-positive compounds. On the other hand, Liming et al. [116] determined 26 amino acids in RJ. This intricate balance of vitamins and amino acids in RJ, along with other food sources, contributes to the overall health and development of honey bees.

Both amino acids and vitamins have been used as food supplements for honey bees [117,118,119]. Glavinic et al. [118] demonstrated that newly emerged worker bees fed a diet supplemented with a combination of vitamins, minerals, and amino acids had a significantly reduced number of Nosema spores compared to the control, particularly on day 12 post-infection. This effect was observed in the context of immunosuppression induced by the microsporidium *Nosema ceranae*. Similarly, Stanimirović et al. [119] demonstrated a significant and consistent increase in the hygienic behavior of honey bees when they were fed with the same diet mentioned above, which can help the colony resist microsporidial and viral infections.

### 2.5. Water

In addition to the nutrient requirements described above, water is also essential for the survival of honey bees. According to Nicolson [120], bees use water not only to maintain osmotic homeostasis but also for several other tasks within the hive, such as cooling the brood area, regulating the hive temperature, and feeding the larval brood, due to the high water content in RJ. Moreover, water can also provide minerals to bees [121]. Mineral nutrients such as sodium (Na), magnesium (Mg), and potassium (K) are essential to meet the nutritional needs of bees [122,123]. According to Khan et al. [124], honey bee colonies showed a preference for sodium chloride (NaCl), potassium chloride (KCl), and magnesium chloride (MgCl_2_) over deionized water in both the summer and winter seasons.

A study conducted by Filipiak et al. [121] suggested that Na deficiencies in honey bee diets must be supplemented using “dirty water”, which is rich in salts [123]. Similarly, Cairns et al. [125] highlighted the importance of stoichiometrically balanced diets for honey bees, suggesting that “dirty water” may be a good source of mixed minerals. However, it is important to ensure that any supplementary sources do not harm the bees. Further studies are needed to better understand honey bee water foraging preferences.

## 3. Conclusions

Considering all the nutritional requirements of *A. mellifera*, including the need for a well-balanced diet, along with the challenges posed by proximity to agricultural crops and exposure to various stressors, maintaining healthy honey bee colonies has become more challenging for beekeepers. As a result, in response to the demand for stronger and more productive colonies, the use of dietary supplementation in beekeeping practices is becoming more common worldwide, especially because the availability of resources is not always possible. Additionally, decisions regarding food supplementation factors, such as landscape use, climatic conditions, seasonal variations, and the presence of environmental stressors, should be considered, to optimize colony health and productivity. Further research needs to be conducted in order to evaluate the long-term effectiveness of food supplementation on honey bee colonies.

## Data Availability

Not applicable.
